# Primary pulmonary Hodgkin’s lymphoma coexisting with extreme erythrocyte sedimentation rate

**DOI:** 10.11613/BM.2024.030802

**Published:** 2024-10-15

**Authors:** Darko Katalinic, Ivan Aleric, Ivana Skrlec, Jasminka Talapko, Elke Kattner, Damir Tentor, Aleksandar Vcev

**Affiliations:** 1 Department of Clinical Medicine, Faculty of Dental Medicine and Health, Josip Juraj Strossmayer University of Osijek, Osijek, Croatia; 2 Department of Medical Biology and Genetics, Faculty of Medicine, Josip Juraj Strossmayer University of Osijek, Osijek, Croatia; 3 Department of Internal Medicine, Faculty of Medicine, Josip Juraj Strossmayer University of Osijek, Osijek, Croatia; 4 Department of Biophysics, Biology and Chemistry, Faculty of Dental Medicine and Health, Josip Juraj Strossmayer University of Osijek, Osijek, Croatia; 5 Department of Integrative Medicine, Faculty of Dental Medicine and Health, Josip Juraj Strossmayer University of Osijek, Osijek, Croatia; 6 Department of Hematology and Medical Oncology, Herford Teaching Hospital, Herford, Germany; 7 Department of Pathology and Cytology, Karolinska University Hospital, Stockholm, Sweden

**Keywords:** blood sedimentation, hematopoietic stem-cell transplantation, lung

## Abstract

The paper aims to present the case of an asymptomatic 22-year-old man who was referred to the hematologist by laboratory experts primarily due to the extreme elevation of the erythrocyte sedimentation rate with a value of 197 mm/h. Additionally, moderate changes in laboratory parameters such as hemoglobin, leukocytes, lactate dehydrogenase, C-reactive protein, fibrinogen, and beta-2-microglobulin were recorded. Upon extensive clinical workup that included laboratory, imaging, and histological methods, a diagnosis of primary pulmonary Hodgkin’s lymphoma (PPHL) was established. Primary pulmonary Hodgkin’s lymphoma is a rare malignant lymphoproliferative disease that exclusively affects the lungs, and so far, only about 100 cases worldwide have been reported. The patient underwent first-line systemic chemotherapy with chest radiation and complete remission was obtained. Two years after completion of the treatment, a relapsed PPHL was clinically confirmed. Second-line chemotherapy followed by high-dose systemic chemotherapy with autologous hematopoietic stem-cell transplantation was indicated which led to complete remission and continues after 10 years from the initial diagnosis. The case demonstrates the important role of laboratory medicine experts who instantly suspected the possible laboratory-related tumor pathology and referred the patient to further hemato-oncological evaluation. This contributed to the timely diagnosis of PPHL, administration of appropriate treatment, and favorable outcome.

## Graphical abstract

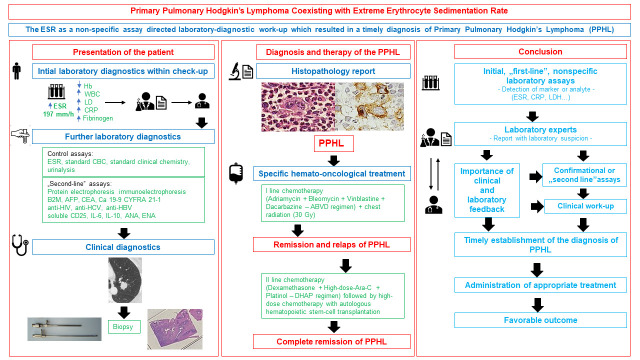

## Highlights

• A case of primary pulmonary Hodgkin’s lymphoma with elevated erythrocyte sedimentation rate (ESR) was evaluated

• The importance of laboratory experts within lab-related pathology is emphasized

• Nonspecific assays like ESR could direct other assays to guide the diagnosis

## Introduction

In the countries of the Western world, the incidence of Hodgkin’s lymphoma (HL) is around 3 *per* 100,000 adults (*1*). Among this group, primary pulmonary Hodgkin’s lymphoma (PPHL), also known as primary pulmonary Hodgkin’s disease, is a rare clinical entity (0.4% of all lymphomas). Until now, only about 100 cases worldwide have been reported (*2*-*4*). It should be distinguished from nodal HL with lung involvement, particularly non-Hodgkin lymphoma as well as pseudo-lymphoma. According to Kern *et al*., the clinical criteria for the diagnosis of PPHL are as follows: specific histological features of HL, limitation of tumor tissue to the lung without lymph node involvement, and adequate clinical and/or pathologic exclusion of tumor spreading at distant sites (*2*). The limitation of disease to the lung parenchyma offers a chance for early treatment and a favorable outcome. We aim to present a rare, unusual case with the clinical course of a patient with a high erythrocyte sedimentation rate (ESR) who was ultimately diagnosed with PPHL upon extensive clinical work-up. Moreover, we also aimed to emphasize the important role of the laboratory experts within the diagnostic procedure with the importance of multidisciplinary cooperation between the laboratory experts and clinicians.

## Case report

**Figure 1:**
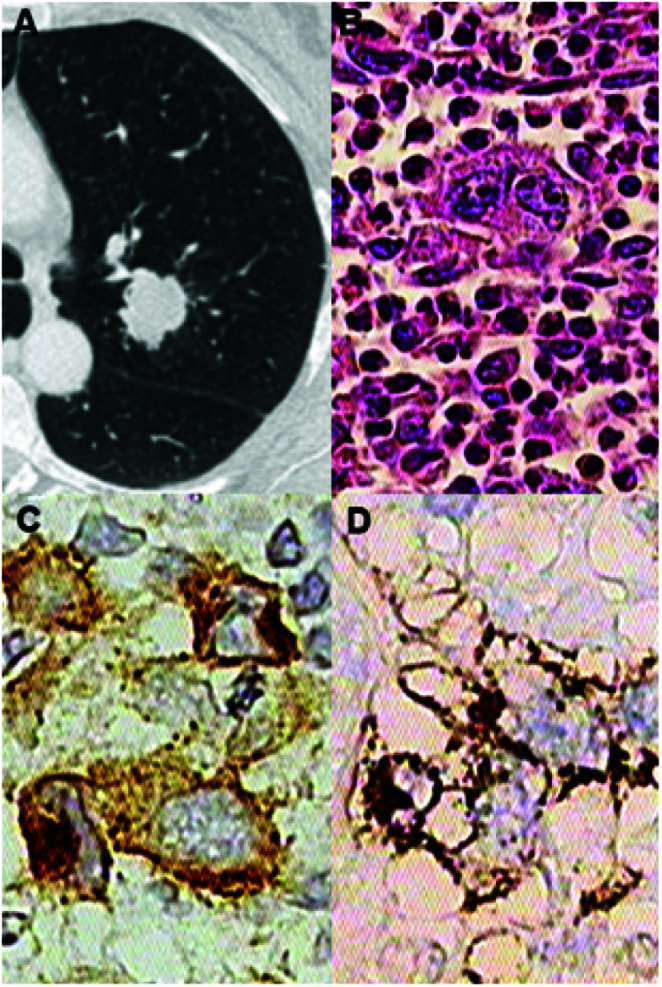
Diagnostic imaging and immunohistochemical staining with histopathological analysis in a patient with primary pulmonary Hodgkin’s lymphoma. A) Contrast-enhanced computed tomography scan of the chest revealed a heterogeneous tumor mass in the left lung. B) Histopathology analysis revealed scattered tumor cells with prominent nucleoli and Reed-Sternberg (RS) cells within a composite inflammatory milieu composed of eosinophils, small lymphocytes, and sclerosis, all consistent with a diagnosis of primary pulmonary Hodgkin’s lymphoma, classical type, nodular sclerosing type 1 (original magnification, 200x). C) and D) Hodgkin and RS cells are immunohistochemically strong positive for CD30 and CD15 (original magnification, 630x).

The main reason for referring a 22-year-old male patient to the hematologist by laboratory experts was the extreme elevation of the ESR with the value of 197 (reference interval 3-23 mm/h). The basic laboratory analyses were carried out as part of the patient’s regular annual medical check-up. They included ESR, standard parameters of complete blood count, coagulogram, clinical chemistry, and urinalysis. Only the values of ESR, hemoglobin, leukocytes, lactate dehydrogenase (LD), C-reactive protein (CRP), and fibrinogen indicated a possible medical occurrence (Table 1). To clarify the background of the basic laboratory findings, *i.e.* the possible neoplastic, autoimmune, or infectious event, the patient was admitted to the Department of Hemato-Oncology one day after initial laboratory screening. He was asymptomatic, without any comorbidity. His physical examination and medical history were normal without any clinical signs of the infection. Therefore, the clinical and laboratory work-up was primarily aimed at detecting an occult neoplasm or an autoimmune process. Immediately after the clinical examination, a control blood sample was taken from the patient for control laboratory assays,“second-line” assays, and microbiology analyses. Control laboratory assays included ESR, standard complete blood count, standard clinical chemistry, and urinalysis. The control results were in concordance with the initial findings (data not shown). The “second-line” assays included protein electrophoresis, immunoelectrophoresis, beta-2-microglobulin (BMG), tumor markers (alpha-fetoprotein, carcinoembryonic antigen, cancer antigen 19-9, cytokeratin fragment 21-1, chromogranin A), virological (anti-HIV, anti-HCV, anti-HBV) and immunological tests (soluble CD25, interleukin 6 and 10, antinuclear antibodies, extractable nuclear antigens). The results of the listed tests were normal (data not shown) except for the value of BMG which was slightly elevated (Table 1). The results of microbiology analyses (blood and urine cultures, sputum smear, and molecular nucleic acid amplification test for tuberculosis) were also normal. During the next two days of hospitalization, a further clinical work-up was carried out, which included electrocardiography, echocardiography, thyroid ultrasound, and pulmonary function tests. The results were normal. As the chest radiograph was inconclusive, a contrast-enhanced computed tomography (CT) scan of the chest was performed and showed evidence of a heterogeneously enhancing soft tissue mass in the left lung which required further clinical clarification (Figure 1A). Fiber-optic bronchoscopy with bronchial washing showed no endobronchial pathology. The metastatic work-up (whole-body CT) scan was negative. However, histological specimens obtained by percutaneous transthoracic CT-guided biopsy of the mass within the left lung were clinically relevant. Microscopic examination revealed scattered tumor cells with prominent nucleoli and Reed-Sternberg cells (Figure 1B) as well as positive immunohistochemical reaction with CD30 and CD15 on the lymphoid cells, all consistent with a diagnosis of PPHL, classical type, nodular sclerosing type 1 (Figure 1C and D). The presence of Epstein-Barr Virus (EBV) in the malignant cells was confirmed by the expression of the EBV-noncoding small viral RNA (EBER1) (data not shown). Histological examination of the bone marrow didn’t reveal tumor infiltration. After reproductive counseling, the patient underwent first-line systemic chemotherapy (Adriamycin + Bleomycin + Vinblastine + Dacarbazine - ABVD regimen) with chest radiation (30 Gy). The complete remission was obtained. Two years after completion of treatment, due to the appearance of B-symptoms (night sweats) and new radiological features, percutaneous transthoracic CT-guided biopsy of the left lung and consecutive histology examination confirmed relapsed PPHL. Second-line chemotherapy (Dexamethasone + High-dose-Ara-C + Platinol - DHAP regimen) followed by high-dose systemic chemotherapy with autologous hematopoietic stem-cell transplantation was indicated which led to a complete regression of the disease, and which continues after 10 years from the initial diagnosis.

**Table 1:** Laboratory parameters of the patients with primary pulmonary hodgkin’s lymphoma on admission and 24 hours upon admission to the Department of Hemato-Oncology

**Parameter** **(on admission)**	**Value**	**Reference interval**
Erythrocyte sedimentation rate (mm/h)	197	3-23
C-reactive protein (mg/L)	12.0	< 5.00
Lactate dehydrogenase (U/L)	325	1-241
Hemoglobin (g/L)	122	138-175
White blood cells (x10^9^/L)	11.1	3.4-9.7
Fibrinogen (g/L)	5.2	1.8-3.5
**Parameter** **(24 hours upon admission)**		
Beta-2-microglobulin (g/L)	2.6	0.8-2.4

## Discussion

Our patient was primarily referred to the hematologist by laboratory experts for clarification of the extreme ESR value. This generated further clinical work-up and resulted in a timely diagnosis of PPHL. Erythrocyte sedimentation rate represents a simple, fast, and inexpensive laboratory test that is frequently ordered in clinical medicine. It can be elevated in many different conditions, but it is not specific to any one disease. The test has been used for over a century, primarily in the diagnostic of acute and chronic inflammatory conditions as well as tumor diseases, although ESR elevation is also found in pregnancy, autoimmune and rheumatic diseases, ischemic stroke, coronary heart disease, chronic kidney failure, and albumin deficiency (*5*). Furthermore, some lifestyle factors (*i.e.* alcohol consumption, smoking) and metabolic abnormalities (*i.e.* diabetes mellitus, overweight, metabolic syndrome) may also influence ESR values (*6*, *7*). In the context of HL, ESR proved to be an old, but valuable marker. Ekek *et al.* and Rafiq *et al.* have demonstrated that increased values of ESR and CRP occur in many patients with HL, sometimes even several months before the definitive diagnosis of HL has been established (*8*, *9*). In addition to elevated ESR and CRP concentration, Rafiq *et al.* also showed that > 70% of patients with HL showed changes in an additional 4 inflammatory markers (prothrombin time, platelets, ferritin, and albumin) (*9*). This shows that a strong inflammatory response is occurring in HL, even in patients without clear clinical symptoms. In our patient, the mentioned tests were within the reference interval, but the number of leukocytes, as well as the concentration of hemoglobin and fibrinogen, were changed. Abnormal concentrations of inflammatory markers can represent early detectable laboratory signs of a malignant process which has significant clinical importance. As in our case, a high ESR can support the diagnosis of HL, especially when combined with other laboratory tests and diagnostic methods such as BMG and LD, as well as radiology imaging and tissue/lymph node biopsy (*10*). Additionally, high ESR values are also associated with spreading lymphoma to distant sites of the body and may indicate a poor prognosis. Staging for HL is based on the Ann Arbor staging system with two modifications in the form of the Cotswolds-modified Ann Arbor classification and the Lugano classification system. According to the European Organisation for the Research and Treatment of Cancer (EORTC), the National Comprehensive Cancer Network (NCCN), and the German Hodgkin’s Study Group (GHSG), the ESR assay is used to allocate early-stage patients to favorable or unfavorable subgroups (*11*).

An ESR value > 30 mm/h (in patients with B symptoms) and > 50 mm/h (in patients without B symptoms) is considered one of the important risk factors of HL and a serological marker that may indicate disease relapse. This is additionally relevant from a therapeutic point of view since the patients without a high ESR are classified in the limited stage and those with a high ESR in the intermediate stage. Moreover, such laboratory data is of great importance because it classifies the patient in another therapeutic category which then directly affects the type of radio/chemotherapy and the extent of treatment (*10*-*13*). Additionally, ESR can be also used to monitor the activity of HL during and after treatment. Decreasing ESR values may indicate a positive response to therapy, while during and after the treatment increasing ESR values could suggest relapse or progression of the HL (*12*). Like ESR, CRP represents a non-specific serum marker, yet can be also clinically important for the follow-up of patients with HL (*14*). It is typically related to inflammation, with the fact that an increase in ESR value is usually accompanied by a parallel increase in CRP. However, in our case, the situation was quite opposite. Interestingly, both initial and control laboratory reports showed only slightly elevated CRP concentrations. In addition to the normal clinical status of the patient and the absence of an inflammatory event, the discordant pattern of ESR and CRP further deepened the suspicion of the existence of an occult, asymptomatic malignant disease. Similar to our study, Aljehani *et al.* presented 3 patients with PPHL in whom CRP concentrations were also lower than ESR values although there were differences concerning clinical symptomatology (*15*). Still, by measuring both parameters, their predictive role in combination with clinical history and physical exam is significantly enhanced (*16*-*18*). Furthermore, in our patient, in addition to high ESR, some other changes in laboratory parameters were also found, which also differentially and diagnostically indicated the possibility of the existence of inflammation or malignancy. This primarily refers to the increased BMG and LD. Beta-2 microglobulin is a ubiquitously expressed cell protein that is primarily involved in the function of the immune system. Elevated concentrations of BMG can be also associated with solid and hematologic malignancies as well as other non-malignant conditions such as liver or renal dysfunctions, and viral infections (*19*). When considering HL, BMG concentrations are used to help assess the disease progression and determine the effectiveness of treatment. As well as ESR and CRP, high concentrations of BMG may indicate a more advanced stage or a poorer prognosis (*20*). A decrease in BMG concentrations during therapy may indicate a good response, whereas stable or increasing concentrations might suggest treatment resistance or relapse. These findings are also supported by the study of Vassilakopoulus *et al.* on 915 patients with HL, who showed that BMG is an independent predictor and adverse prognostic factor of outcome in HL (*21*). Lactate dehydrogenase is an enzyme that plays a key role in the body’s metabolic processes. Its activity can be important in various medical conditions, including HL. In addition to ESR, CRP, and BMG, it is important to note that LD activity is also a nonspecific marker and can be elevated in many other conditions, such as other types of cancer, hemolysis, liver disease, and infections. High activity of LD can be indicative of tissue breakdown, which is often seen in different cancerous conditions. Activity of LD can be also monitored over time to assess the effectiveness of treatment. A decrease in LD activity during treatment might suggest a good response, whereas persistently high or increasing activity could indicate advanced stage, refractory disease, or relapse (*10*, *22*).

When we consider PPHL, establishing a precise diagnosis and administration of proper therapy in a short time can be challenging for clinicians, especially considering the multiple procedures that are required. Although rare, it is possible that patients with PPHL are asymptomatic at initial presentation, and disease is discovered by accident on screening chest X-ray or the laboratory experts notice unusual values of laboratory findings. While ESR as well as CRP, BM2, and LD are nonspecific, they are very useful adjuncts in the management of HL, and always need to be contextualized alongside other diagnostic findings to guide proper decision-making. The core of the diagnosis of PPHL is and will remain the histopathological analysis of an excisional biopsy or a surgical specimen accompanied by proper imaging methods. However, the present case illustrates the importance of laboratory findings, for both laboratory experts and clinicians, and considering malignant lesions in the differential diagnosis even in patients in whom such a diagnosis is unlikely. Finally, although significantly more sensitive and specific methods than ESR are used in laboratory practice, it is also necessary to emphasize that simple, and cheap laboratory tests should never be completely “diagnostically forgotten” because they sometimes can very well direct further diagnostic and therapeutic approaches and complete the diagnostic puzzle.

The ESR measurement and interpretation of the laboratory and clinical results have potential limitations. They are manifested in methods/instruments/techniques used to collect and analyze the blood sample as well as in laboratory-clinical characteristics of the patient. Furthermore, the limitation also includes the elements of the clinical treatment of the patient, which incorporates the lack of information, misinterpretation, or insufficient monitoring of certain data from the anamnesis or clinical examination. All this can significantly affect the direction of further diagnostic treatment and the selection of diagnostic tests. Also, the determination of ESR as a non-specific laboratory assay can certainly help in directing similar patients in which there is a suspicion of the existence of lymphoma for further diagnostic processing, but it needs to be considered the limitation of the test in establishing a final diagnosis, *i.e.* its significant shortcoming in the light of its low specificity.

## Data Availability

All data generated and analyzed in the presented study are included in this published article.

## References

[1] Weniger MA Küppers R. Molecular biology of Hodgkin lymphoma. Leukemia. 2021;35:968–81. 10.1038/s41375-021-01204-6 33686198 PMC8024192

[2] Kern WH Crepeau AG Jones JC. Primary Hodgkin’s disease of the lung. Report of four cases and review of the literature. Cancer. 1961;14:1151–65. 10.1002/1097-0142(196111/12)14:6<1151::AID-CNCR2820140604>3.0.CO;2-1 14455298

[3] Kumar R Sidhu H Mistry R Shet T. Primary pulmonary Hodgkin’s lymphoma: A rare pitfall in transthoracic fine needle aspiration cytology. Diagn Cytopathol. 2008;36:666–9. 10.1002/dc.20872 18677750

[4] Jung H Kim HS Han J Ko YH Choi YD Lee T. Clinicopathological Characteristics of Primary Pulmonary Hodgkin Lymphoma (PPHL): Two Institutional Experiences with Comprehensive Literature Review of 115 PPHL Cases. J Clin Med. 2022;12:126. 10.3390/jcm12010126 36614926 PMC9821715

[5] Erythrocyte Sedimentation Rate. Tishkowski K, Gupta V. Available from: https://www.ncbi.nlm.nih.gov/books/NBK557485/. Accessed May 30th 2024. 32491417

[6] Brigden ML. Clinical utility of the erythrocyte sedimentation rate. Am Fam Physician. 1999;60:1443–50. 10524488

[7] Alende-Castro V Alonso-Sampedro M Vazquez-Temprano N Tuñez C Rey D García-Iglesias C Factors influencing erythrocyte sedimentation rate in adults: New evidence for an old test. Medicine (Baltimore). 2019;98:e16816. 10.1097/MD.0000000000016816 31441853 PMC6716712

[8] Ekek ET Bozkurt OF Evman MD. Erythrocyte Sedimentation Rate May Predict Diagnosis of Lymphoma Without Fine-needle Aspiration Biopsy: A Retrospective Study. Bagcilar Med Bull. 2021;6:190–7. 10.4274/BMB.galenos.2021.12.083

[9] Rafiq M Abel G Renzi C Lyratzopoulos G. Inflammatory marker testing in primary care in the year before Hodgkin lymphoma diagnosis: a UK population-based case-control study in patients aged ≤50 years. Br J Gen Pract. 2022;72(721):e546–55. 10.3399/BJGP.2021.0617 35817582 PMC9282809

[10] Ahmed R Tariq F Ashfaq J Thakur W Zafar S Danish A The Outcome of Hodgkin Lymphoma With Reference to Prognostic Markers. Cureus. 2022;14:e28421. 10.7759/cureus.28421 36176827 PMC9512312

[11] Klimm B Goergen H Fuchs M von Tresckow B Böll B Meissner J Impact of risk factors on outcomes in early-stage Hodgkin’s lymphoma: an analysis of international staging definitions. Ann Oncol. 2013;24:3070–6. 10.1093/annonc/mdt413 24148816

[12] Munir F Hardit V Sheikh IN AlQahtani S He J Cuglievan B Classical Hodgkin Lymphoma: From Past to Future—A Comprehensive Review of Pathophysiology and Therapeutic Advances. Int J Mol Sci. 2023;24:10095. 10.3390/ijms241210095 37373245 PMC10298672

[13] Eichenauer DA Aleman BMP André M Federico M Hutchings M Illidge T ESMO Guidelines Committee. Hodgkin lymphoma: ESMO Clinical Practice Guidelines for diagnosis, treatment and follow-up. Ann Oncol. 2018;29:iv19–29. 10.1093/annonc/mdy080 29796651

[14] Negreiros EADS da Silveira TMB Fortier SC Chiattone CS. Evaluation of C-reactive protein and its prognostic relationship in patients with Hodgkin’s Lymphoma. Hematol Transfus Cell Ther. 2024:S2531-1379(24)00003-8. 10.1016/j.htct.2023.11.011 38307826

[15] Aljehani Y Al-Saif H Al-Osail A Al-Osail E. Multiloculated Cavitary Primary Pulmonary Hodgkin Lymphoma: Case Series. Case Rep Oncol. 2018;11:90–7. 10.1159/000486824 29606947 PMC5869374

[16] Cennamo M Giuliano L Arrigoni G Fardone V Russo R De Tomasi LM Method Comparison of Erythrocyte Sedimentation Rate Automated Systems, the VES-MATIC 5 (DIESSE) and Test 1 (ALIFAX), with the Reference Method in Routine Practice. J Clin Med. 2024;13:847. 10.3390/jcm13030847 38337540 PMC10856312

[17] Alende-Castro V Alonso-Sampedro M Fernández-Merino C Sánchez-Castro J Sopeña B Gude F C-Reactive Protein versus Erythrocyte Sedimentation Rate: Implications Among Patients with No Known Inflammatory Conditions. J Am Board Fam Med. 2021;34:974–83. 10.3122/jabfm.2021.05.210072 34535522

[18] Bray C Bell LN Liang H Haykal R Kaiksow F Mazza JJ Erythrocyte Sedimentation Rate and C-reactive Protein Measurements and Their Relevance in Clinical Medicine. WMJ. 2016;115:317–21. 29094869

[19] Li L Dong M Wang XG. The Implication and Significance of Beta 2 Microglobulin: A Conservative Multifunctional Regulator. Chin Med J (Engl). 2016;129:448–55. 10.4103/0366-6999.176084 26879019 PMC4800846

[20] Mumtaz T Iqbal MA Roohi N Akhtar MW. Increased Turnover of Beta 2 Microglobulin in Circulation serves as Diagnostic and Prognostic Marker for Malignant Lymphoma; A Case Control Predictive Model for Lymphoma Diagnosis: Beta 2 microglobulin as a diagnostic and prognostic marker. PJHS. 2023;4:160–5. 10.54393/pjhs.v4i05.757

[21] Vassilakopoulos TP Arapaki M Diamantopoulos PT Liaskas A Panitsas F Siakantaris MP Prognostic Impact of Serum β2-Microglobulin Levels in Hodgkin Lymphoma Treated with ABVD or Equivalent Regimens: A Comprehensive Analysis of 915 Patients. Cancers (Basel). 2024;16:238. 10.3390/cancers16020238 38254729 PMC10813286

[22] Tisi MC Salvini M Federico M. The prognostic role of serum biomarkers in Hodgkin lymphoma. Clin Lymphoma Myeloma Leuk. 2018;18:353–9.29610029

